# Prolonged apnea in a boy with epilepsy and a novel gain-of-function missense CACNA1A variant indicating SUDEP risk

**DOI:** 10.3389/fneur.2025.1582548

**Published:** 2025-07-09

**Authors:** Simone Pelizzari, Marta Campiglio, Yousra El Ghaleb, Tatjana Bierhals, Maja Hempel, Jonas Denecke, Bernhard E. Flucher, Jessika Johannsen

**Affiliations:** ^1^Department of Physiology and Medical Physics, Innsbruck Medical University, Innsbruck, Austria; ^2^Institute of Human Genetics, University Medical Center Hamburg-Eppendorf, Hamburg, Germany; ^3^Institute of Human Genetics, University Heidelberg, Heidelberg, Germany

**Keywords:** P/Q-type calcium channels, CaV2.1, status epilepticus, brainstem spreading depolarization, preventive strategies

## Abstract

**Introduction:**

The *CACNA1A* gene encodes the pore-forming subunit of the Cav2.1 (P/Q type) neuronal calcium channel and pathogenic variants cause a variety of neurological disorders including episodic and congenital ataxia, familial hemiplegic migraine, developmental delay and epilepsy. Multiple types of seizures have been described in affected patients, including status epilepticus as the first manifestation. In mice harboring the homozygous gain-of-function variant p.Ser218Leu, seizures leading to SUDEP triggered by brainstem spreading depolarization with subsequent apnea and cardiac arrest have been reported.

**Methods:**

Clinical, genetic and functional data are presented.

**Results and discussion:**

The 9-year-old boy with global developmental delay and congenital ataxia developed recurrent seizures and status epilepticus with prolonged, life-threatening apnea implying a high risk for SUDEP. Genetic testing showed a novel *de novo* missense variant in *CACNA1A* (c.5398T>A, p.Phe1800Ile). Functional analysis revealed a gain of channel function as the molecular pathomechanism. Therefore, an increased risk of SUDEP in patients with *CACNA1*-associated epilepsy seems reasonable and preventive strategies should be discussed with caregivers.

## 1 Introduction

The *CACNA1A* gene encodes the pore-forming α_1_A subunit of the neuronal voltage-gated calcium channel Cav2.1 (P/Q type). The Ca_v_2.1 channel consists of four homologous domains, each comprising six transmembrane helices (S1-S6) connected by intra- and extracellular loops. Segments S1-S4 of each repeat form the voltage sensing domains, segments S5-S6 of all four repeats together form the conduction pore with the selectivity filter and the activation gate. In the central and peripheral nervous system, Ca_v_2.1 plays a key role in initiating neurotransmitter release in excitatory and inhibitory synapses and is involved in intracellular signaling, transcriptional regulation and neuronal viability (1–4). Pathogenic variants of the *CACNA1A* gene result in allelic disorders with a broad clinical spectrum including episodic ataxia type 2 (OMIM: 108500), familial hemiplegic migraine type 1 (OMIM: 141500), spinocerebellar ataxia type 6 (OMIM: 183086) (5, 6), and overlapping features between these conditions in individual patients. In addition, *CACNA1A*-related epilepsy (OMIM: 617106) with multiple seizures types and status epilepticus have been described (7–10). Sudden unexpected death in patients with epilepsy (SUDEP) is a rare but significant risk in the cohort of patients with epilepsy of heterogeneous etiology. Brainstem spreading depolarization has been discussed to play an important role, possibly leading to brainstem dysfunction followed by respiratory and cardiac arrest (11–15). Variants in *CACNA1A* have been found to be one of the susceptibility genes to SUDEP (16).

Here, we report a 9-year-old boy who initially presented with global developmental delay and congenital ataxia caused by a novel *de novo* missense variant in *CACNA1A*. Functional analysis of the variant revealed altered gating properties leading to a predominantly gain of function phenotype. Epilepsy began with recurrent status epilepticus after the age of 6 years. The seizures were followed by prolonged and life-threatening central apneas, constituting a high risk of sudden unexpected death.

## 2 Materials and methods

The patient charts were reviewed for the clinical history, the laboratory (including metabolic and genetic) and radiological investigations. Blood samples from the patient and his parents were obtained after informed consent. Whole exome sequencing was performed after written informed consent according to national regulations on genetic diagnostics.

### 2.1 Whole exome sequencing and data analysis

DNA samples from whole blood were isolated by standard procedures. Trio whole-exome sequencing (trio-WES) was performed with DNA samples of both healthy parents and the index patient, as described previously (17).

The functional impact of the identified variants was predicted using CADD, REVEL, M-CAP *in silico* tools (18–21).

### 2.2 Plasmids

To generate the GFP-Ca_V_2.1 expression plasmid, the cDNA sequence of human Ca_V_2.1 (nt 1–676) was amplified in separate PCR reactions using pβA-Ca_V_2.1 [GenBankTM FJ040507; (22)] as template with a primer introducing a SalI site at the 5’end. The obtained PCR product was then SalI/NotI digested, the remaining cDNA sequence coding for Ca_V_2.1 was isolated from pβA-Ca_V_2.1 by NotI/BamHI digestion and the two fragments were ligated into the corresponding sites of GFP-Ca_V_1.2 (23).

To generate the GFP-Ca_V_2.1-F1800I expression plasmid, the F1802I mutation in GenBank™ FJ040507 (corresponding to F1800I in the CACNA1A variant GenBank™ AAB64179.1) was introduced into GFP-Ca_V_2.1 by splicing by overlap extension (SOE) PCR. For simplicity reasons, henceforth we will be using the name Ca_V_2.1-F1800I. Briefly, the cDNA sequence of human Ca_V_2.1 (nt 5,072–5,980) was amplified in separate PCR reactions using GFP-2.1 as template with overlapping primers mutating the c.5404T>A. The two separate PCR products were then used as templates for a PCR reaction with flanking primers to connect the nucleotide sequences. The resulting fragment was then XhoI/BglII digested and ligated into the corresponding sites of GFP-Ca_V_2.1.

All newly generated sequences were verified by sequencing (Eurofins genomics).

### 2.3 Electrophysiology

The experiments were conducted on an in-house produced cell line (A2MG), a HEK 293 cell line that stably expresses human β3 and α2δ-1 calcium channel subunits (24, 25). Calcium currents were recorded using the ruptured whole-cell patch-clamp technique in voltage-clamp mode. Patch pipettes (borosilicate glass, Harvard Apparatus, Holliston, MA) had a resistance of 2.5–4.5 MΩ when filled with a solution containing (mM) 135 CsCl, 1 MgCl2, 10 HEPES, 4 ATP-Na2, and 10 EGTA (pH 7.4 with CsOH). 10 mM concentration of EGTA prevents calcium-dependent inactivation (CDI) (26). The extracellular bath solution comprised (mM) 15 CaCl2, 150 choline-chloride, 10 HEPES, and 1 MgCl2 (pH 7.4 with CsOH). All experimental groups were analyzed in transiently transfected cells from three to six independent cell passages. For each cell, the stimulation protocol was only recorded once (no technical replicates). The recordings were acquired with Axopatch 200A amplifier (Axon Instruments, Foster City, CA). Data acquisition and command potentials were controlled by pClamp software (version 10.7, Axon Instruments). Current–voltage (I–V) relationships were obtained by applying a voltage-step square pulse protocol starting from a holding potential (Vhold) of −80 mV followed by the command potential (Vcmd) of 500 ms, ranging from −60 mV to +80 in 10 mV increment. The resulting I–V curves were fitted to the following equation:


(1) $$I={\text{Gmax}}^{*}(V–\text{Vrev})/(1+\exp(–(V–V1/2)/K))$$

Where Gmax is the maximum conductance of the calcium channels, Vrev is the extrapolated reversal potential of the current, V1/2 is the potential for half-maximal conductance, and k is the slope.

The conductance was extrapolated using:


(2) $$G=(–I^{*}1000)/(\text{Vrev}–\text{Vcmd})$$

The conductance voltage dependence was calculated according to the Boltzmann distribution:


(3) G=Gmax/(1+exp(–(V–V1/2)/K))

The time constant of activation (*τ*_act_) was obtained by applying a mono-exponential fit to the rising phase of the current using the equation:


(4) F(t)=A×(1–exp(−t/τ))

In contrast, the time constant of inactivation (*τ*_inact._) was obtained by fitting the decay phase of the current with a mono-exponential function described by:


(5) F(t)=A×(exp(−t/τ))

For both equations, A is the current amplitude, and τ corresponds to the time constant of either activation or inactivation.

The voltage dependence of inactivation was adapted from Gambeta et al. (27) by application of two test pulses to V_max_ (at +20 mV for WT and at +10 mV fore F1800I) before and after holding cells at various conditioning test potentials (ranging from −80 to +50 mV) for a duration of 5 s (60 s intersweep interval). Inactivation was calculated as the ratio between the current amplitudes of the test pulses. Steady-state inactivation parameters were obtained by fitting the data to the modified Boltzmann equation, as follows:


(6) $$G=(1–G_{\mathrm{ni}})/((1+\exp((V–V1/2_{,\text{inact}}.)/\text{Kinact})+G_{\mathrm{ni}})$$

Where V1/2_,inact._ is the half-maximal inactivation voltage, kinact is the inactivation slope factor and G_ni_ is the fraction of non-inactivating current in steady-state.

The kinetics of recovery from inactivation was assessed by application of a 5-s-long pre-pulse followed by a test pulse both at V_max_. The test pulse was recorded at various time points (between 20 ms and 45 s, using a logarithmic increase) after the 5 s pre-pulse (30 s intersweep interval). Per each sweep, the rate of recovery from inactivation was calculated as the ratio between the Imax obtained during the test pulse and the Imax collected during the pre-pulse. The rate of recovery from inactivation was best fit using a double exponential equation.

### 2.4 Statistical analysis

SigmaPlot (version 12.0; SPSS) was used for statistical analyses and curve fitting; GraphPad Prism (version 8.0.1; Graphpad Softaware LLC) and CorelDRAW2021 (version 23.0.0.363; Corel Corporation) were used to make the figures. All data are presented as mean ± SEM. First, all the data were assessed for the normality of the distribution using a Shapiro–Wilk test with significant criteria alpha = 0.05. Statistical comparison of the fit parameters were obtained by using either Student’s t test or mixed-effects analysis matched with Šídák’s multiple comparisons test, with significance criteria, ∗≜p<0.05,∗∗≜p<0.01,∗∗∗≜p<0.001,∗∗∗∗≜p<0.0001 .

## 3 Results

Clinical and genetic data are summarized in Table 1.

**Table 1:** Clinical and genetic data of our patient.

**genetic data**	results
**genomic position**	c.5398T>A
**aminoacid change**	p.Phe1800Ile
**protein domain**	domain IV, segment S6
**mode of inheritance**	de novo
**general data**	
**ethnicity**	caucasian / german
**gender**	male
**gestational age (weeks)**	40+5
**pregnancy, birth**	uneventful, spontaneous delivery
**Apgar score**	9/10/10
**birth weight (g) (perc.)**	3340 (P21)
**birth height (cm) (perc.)**	56 (P91)
**birth head circumference (cm) (perc.)**	35.5 (P41)
**age at first examination (months)**	23
**age at latest examination (years)**	9
**weight at latest examination (kg) (perc.)**	43.9 (P89)
**height at latest examination (cm) (perc.)**	144.5 (P65)
**head circumference**	macrocephalus after the age of 4 yrs.
**head circumference at latest examination (cm) (perc.)**	57 (P99)
**clinical findings**	
**dysmorphism**	none
**first symptoms**	muscular hypotonia
**further neurological symptoms**	ataxia, dysarthria
**development**	
**motor development** **age of free sitting (months)** **age of walking (months)**	delayed 12 30
**speech development** **age of first words (months)**	delayed 17
**cognitive development**	moderate intellectual deficits
**school performance**	special needs school
**behavioural disturbances**	no
**epilepsy**	
**age at seizure onset (years)**	6
**seizure types**	focal, focal to bilateral tonic clonic
**febrile seizures**	yes
**Status epilepticus**	yes
**complications after status epilepticus**	encephalopathy, apnea
**postictal EEG findings**	focal slowing over posterior regions
**interictal EEG findings (including 24-hour-EEG)**	normal
**current antiepileptic drugs**	levetiracetam, lamotrigine, topiramate
**further antiepileptic drugs (discontinued)**	acetacolamide, zonisamide, ethosuximide
**further diagnostics**	
**MRI (incl. spectroscopy) at 11 months, 4, 6 and 7 years**	normal
**echocardiography**	normal
**audiometry**	normal
**eye examination**	normal
**metabolic work-up including amino acids (blood, CSF), acylcarnitine profile (blood), organic acids (urine), isoelectric transferrin focusing (blood), glycosaminoglycans (urine)**	normal

### 3.1 Patient data

The 9-year-old boy was born after an uneventful pregnancy as the first child of healthy, unrelated Caucasian parents. At birth, his weight, length and head circumference were within the normal range. A global developmental delay was evident in the boy from infancy onward, and ataxia manifested when he started walking at 27 months of age. His speech was slurred but he learned to speak complete sentences. On neurological examination at the age of 30 months, ataxia and dysarthria were the prominent features. Head circumference became macrocephalic (+2z) after the age of 4 years. At the age of 9 years, the boy shows moderate developmental delay in speech and learning.

The first brain MRI was performed at 11 months of age, and was unremarkable. Subsequent brain MRI including MR spectroscopy at the age of 4, 6, and 7 years were also normal. At the age of 3 years and 9 months, a comprehensive metabolic work-up in blood, urine and cerebrospinal fluid as well as EEG showed no abnormalities, but whole-exome-sequencing revealed the novel *de novo* missense variant [c.5398T>A, p.(Phe1800Ile)] in the *CACNA1A* gene which was consistent with the patient’s symptoms. Treatment with acetazolamide was initiated due to the known positive effects of carbonic anhydrase inhibitors in cases of *CACNA1A*-associated ataxia (28), but was discontinued shortly thereafter because of diarrhea.

After an uneventful period without seizures, headache or hemiplegic episodes, the boy was admitted at the age of 6 years and 2 months with the first generalized, tonic–clonic status epilepticus that was followed by a prolonged period of more than 1 week with somnolence, confusion and inability to speak, sit or walk. Treatment with levetiracetam was initiated despite absence of epileptic discharges on EEG. MRI revealed no abnormalities. The boy recovered over the next few months, slowly regaining all of his former abilities. During the next 17 months, the boy was admitted to the emergency department with multiple focal to bilateral tonic–clonic status epileptici and seizures followed by prolonged central apnea up to 50 min, bradycardia, and hypothermia requiring intubation and mechanical ventilation. The apnea was not on the consequence of benzodiazepine use, as it occurred in part before the administration of a dose of benzodiazepine and was not accompanied by convulsions or increased muscle tone. Postictal EEG showed focal slowing over posterior regions. Several interictal EEGs, including a 24-h EEG, showed normal background activity and no epileptic discharges. After the first status epilepticus, levetiracetam was started, and because of recurrent seizures, the treatment was changed to multiple antiepileptic drugs including carboanhydrase inhibitors, and calcium channel inhibitors, i.e., levetiracetam plus zonisamide, levetiracetam plus ethosuximide, levetiracetam plus ethosuximide plus lamotrigine, and levetiracetam plus lamotrigine plus topiramate, respectively. The latter resulted in a stable situation with only single seizures with mainly spontaneous termination and without postictal apnea. After the first status epilepticus with prolonged and life-threatening postictal apnea, the patient’s risk of dying during seizures seemed extremely high. Therefore, pulse oximetry monitoring during sleep was initiated. At the time of the most recent visit, the boy had been seizure-free for 4 months and had not experienced episodes of severe headache or hemiplegia.

### 3.2 Genetic data

Using WES, we identified the novel *de novo* missense variant c.5398T>A, [p.(Phe1800Ile)] in *CACNA1A* (RefSeq accession number NM_001127221.2). The detected alteration had not been reported in the dbSNP^1^ or GnomAD^2^ databases and was computationally predicted to be functionally relevant with the following scores—CADD: 27.7, REVEL: 0.911 and M-CAP: 0.629. The affected Phe1800 is highly conserved across species and is located within the transmembrane segment S6 of the domain IV, which is part of the channel conduction pore and near a hot-spot for disease-associated variants in calcium channels (29). According to the scoring criteria of American College of Medical Genetics and Genomics (ACMG) this variant was evaluated as pathogenic [applied criteria: PS2, PM2, PP3 and PS3 (see below)] (30). No other candidate genes or known disease genes have been identified that could contribute to the patient’s phenotype.

### 3.3 Functional data

**Figure 1:**
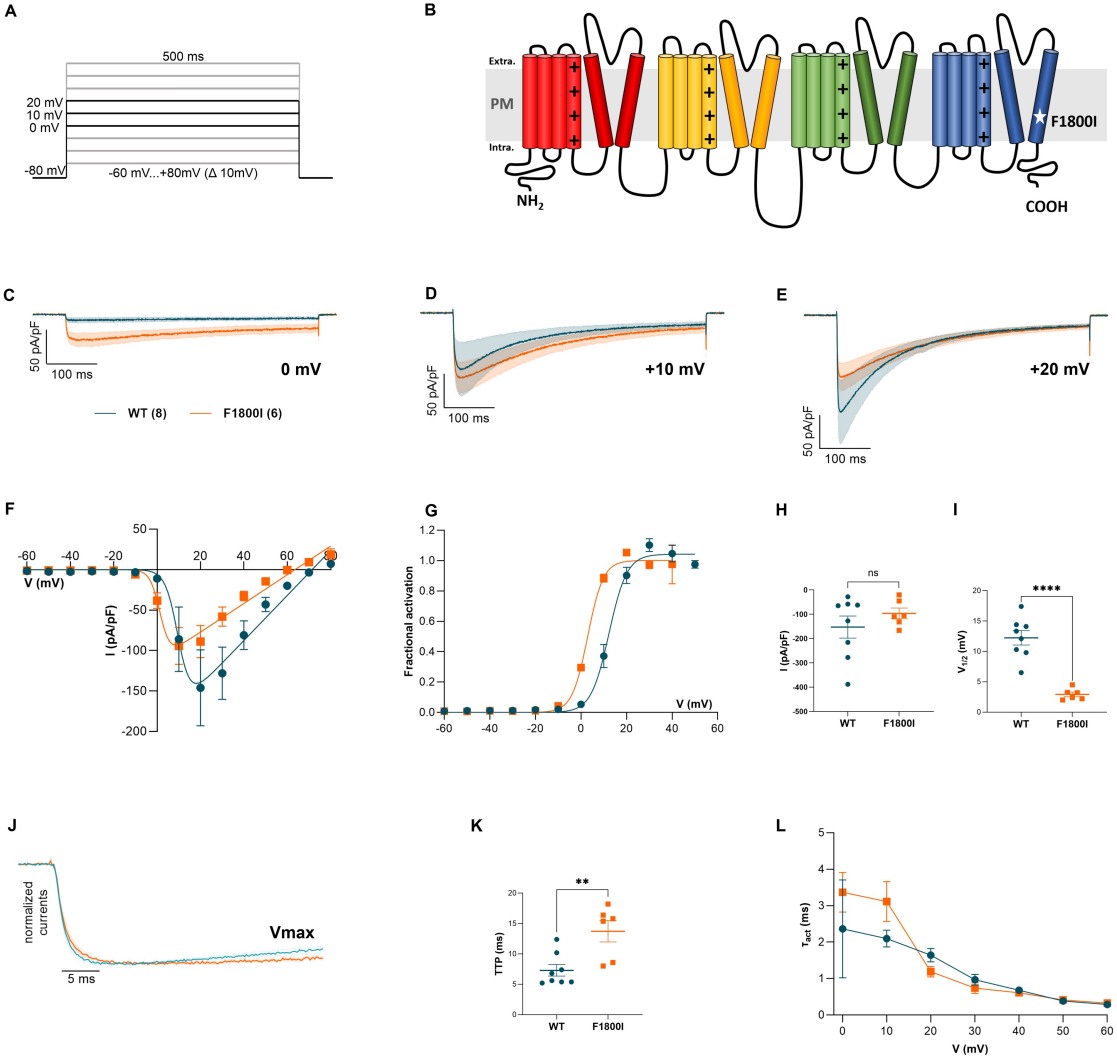
Functional analysis of the Ca_V_2.1-F1800I variant heterologously expressed in HEK-293 cells—Current activation. **(B)** Domain structure of the Ca_V_2.1 α_1_ subunit with the approximate location of the F1800I mutation in the IVS6 gate-forming helix. **(A)** Voltage-clamp protocol. **(C–E)** Calcium currents (mean ± SEM) of wildtype Ca_V_2.1 (WT, blue) and mutant Ca_V_2.1-F1800I (F1800I, orange) in response to 500-ms voltage steps to 0 mV **(C)**, +10 mV **(D)** and +20 mV **(E)** demonstrates that currents are elicited at lower potentials in the disease variant. **(F,G)** I/V curves and fractional activation curves showing a 9.3 mV left-shift of the V_1/2_ of activation for F1800I relative to its control. **(H,I)** Scatter plots of current density and the voltage of half-maximal activation (**H**: *p* = 0.3; **I**: *p* < 0.0001). **(J)** Normalized calcium currents at V_max_ (+20 or +30 mV for WT; +10 or +20 mV for F1800I) show the relative slowing of activation kinetics of the mutant (F1800I, orange) compared to the wild type (WT, blue). **(K)** Scatter plot of the time to peak obtained at V_max_ displaying a significantly slowed activation kinetics of F1800I compared to its control (*p* = 0.005). **(L)** Time constants of current activation calculated at voltage steps (τ_act_) between 0 and +60 mV indicate no significant difference (*p* = 0.1). Mean ± SEM; mutant and WT controls compared by t-tests or mixed-effects analysis matched with Šídák’s multiple comparisons test, using significance criteria, ∗≜p<0.05,∗∗≜p<0.01,∗∗∗≜p<0.01,∗∗∗∗≜p<0.01. .

To examine the effects of the Phe1800Ile substitution on the properties of the Ca_V_2.1 calcium channel we introduced the corresponding mutation in the human Ca_V_2.1 variant (GeneBank™ FJ040507). The domain model in Figure 1B shows the position of the mutation in the S6 helix of domain IV, which forms part of the activation gate. The expression plasmids coding for the wildtype and mutant channel N-terminally tagged with green fluorescent protein (GFP-Ca_V_2.1, GFP-Ca_V_2.1-F1800I) were heterologously expressed in HEK 293 cells. Using the patch-clamp technique, whole-cell calcium currents in response to 500 ms voltage steps from a holding potential of −80 mV to varying test potentials were recorded (Figures 1A,C–E). While peak amplitudes of the calcium currents were not significantly different in wildtype and mutant constructs (Figures 1F,H), the voltage-dependence of activation of Ca_V_2.1-F1800I was shifted by 10 mV to less depolarized potentials (Figures 1F,G,I; Table 2). Consequently, cells expressing the disease-associated variant Ca_V_2.1-F1800I experienced calcium influx at 0 mV, a membrane potential at which wildtype Ca_V_2.1 channels barely opened (Figure 1C). Comparing normalized currents indicated slowed activation kinetics of the Ca_V_2.1-F1800I variant (Figure 1J). Indeed, the time-to-peak measured in the maximal current traces (V_max_) was significantly slower in Ca_V_2.1-F1800I compared to wildtype control (Figure 1K). However, the time constants of current activation measured at the test potentials between 0 and +60 mV revealed no statistical significant difference (Figure 1L).

**Table 2:** Current properties of WT and F1800I mutant Ca_V_2.1.

	**Ca**_**V**_ **2.1 WT**	**F1800I**	*p*-value
**Activation**			
*n*	8	6	
ICa (pA/pF)	-152.7 ± 45.5	-96.4 ± 22.2	0.34
V_0.5_ (mV)	12.2 ± 1.2	2.9 ± 0.4	< 0.0001
TTP (ms)	7.3 ± 0.9	13.7 ± 1.8	0.005
% inact. at 100 ms	47.5 ± 4.4	26.2 ± 4.4	0.006
% inact. at 250 ms	77.5 ± 3.0	60.3 ± 5.5	0.01
% inact. at 500 ms	88.7 ± 1.9	76.3 ± 4.8	0.02
**Steady state inactivation**			
*n*	9	7	
V_0.5_ (mV)	-13.7 ± 1.9	-24.7 ± 3.0	0.006
**Time constants of activation (**τ_act_/ms**)**			
*n*	8	6	
at 0 mV	2.4 ±1.3	3.4 ± 0.5	0.9947
at 10 mV	2.1 ± 0.2	3.1 ± 0.5	0.6277
at 20 mV	1.6 ± 0.2	1.2 ± 0.1	0.4283
at 30 mV	0.9 ± 0.1	0.7 ± 0.1	0.9162
at 40 mV	0.7 ± 0.1	0.6 ± 0.1	0.9976
at 50 mV	0.4 ± 0.1	0.4 ± 0.1	>0.9999
**Time constants of activation (**τ_inact_/ms**)**			
*n*	8	6	
at 0 mV	584.1 ±174.8	572.9 ± 222.0	>0.9999
at 10 mV	260.8 ± 40.9	267.9 ± 52.5	>0.9999
at 20 mV	149.1 ± 25.5	194.8 ± 18.9	0.7439
at 30 mV	123.1 ± 10.1	235.9 ± 36.5	0.1682
at 40 mV	140.4 ± 9.5	233.1 ± 30.7	0.1811
at 50 mV	166.1 ± 15.9	347.9 ± 53.1	0.1558
at 60 mV	190.4 ± 14.5	317.9 ± 63.8	0.6455

**Figure 2:**
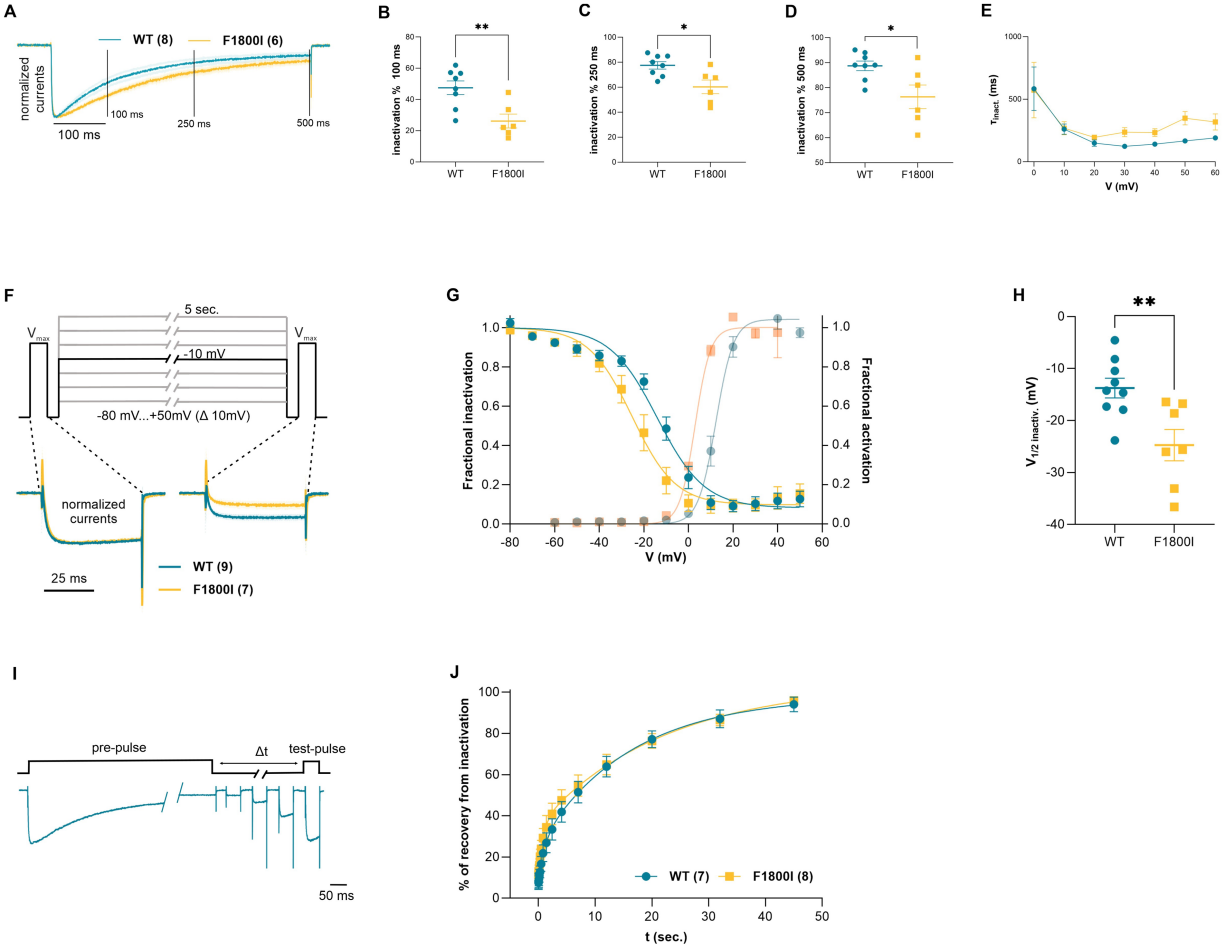
Functional analysis of current inactivation of the Ca_V_2.1-F1800I variant. **(A)** Normalized current traces at V_max_ (blue, wild type, orange, Ca_V_2.1-F1800I; mean ± SEM) showing different time courses of inactivation. The three vertical lines (100, 250 and 500 ms) indicate the specific time points at which fractional inactivation was calculated. **(B–D)** Percent reduction of peak currents at V_max_ after 100, 250 or 500 ms of depolarization is significantly less for F1800I compared with WT (**B**: *p* = 0.006; **C**: *p* = 0.012; **D**: *p* = 0.02). **(E)** Time constant of current inactivation calculated at voltage steps (τ_inact_.) between 0 and +60 mV reveal no statistically significant effect of the mutation on inactivation kinetics (*p* = 0.7). **(F)** Steady state inactivation protocol and typical example traces at −10 mV sweep for Ca_v_2.1 WT (cyan) and Ca_v_2.1-F1800I (yellow). **(G,H)** Fractional inactivation curves and scatter plot of the V_1/2_ of inactivation showing a 10.9 mV shift to more hyperpolarized potential of F1800I compared to WT. Mean ± SEM (**C**: *p* = 0.006). **(I)** Pulse protocol and representative current traces for analyzing the recovery of inactivation (∆t 20 ms to 45 s) after a 5 s. pre-pulse to V_max_ (+20 mV for WT; +10 mV for F1800I). **(J)** The time course of recovery from inactivation does not reveal any significant difference between WT and variant (*p* = 0.58). Mean ± SEM; mutant and WT controls compared by t-tests or mixed-effects analysis matched with Šídák’s multiple comparisons test, using significance criteria, ∗≜p<0.05,∗∗≜p<0.01,∗∗∗≜p<0.01,∗∗∗∗≜p<0.01. .

Mean current densities (ICa), activation kinetics (time to peak, TTP) and percentage of inactivation at 100, 250, and 500 ms were calculated at V_max_ (WT + 20 or +30 mV; F1800I + 10 or +20 mV, respectively). The voltage-dependence of activation (V_0.5_), tau of activation and inactivation (τ_act_, τ_inact._) were measured from voltage-step protocols (Figure 1E). The voltage-dependence of inactivation was determined using a steady-state inactivation protocol (Figure 2A). Mean ± SEM; mutant and WT controls compared by t-tests or mixed-effects analysis matched with Šídák’s multiple comparisons test, using significance criteria, ∗≜p<0.05,∗∗≜p<0.01,∗∗∗≜p<0.01,∗∗∗∗≜p<0.01.

The normalized current traces at V_max_ also indicate a slowed inactivation of the Ca_V_2.1-F1800I currents (Figure 2A). Accordingly, the fractional inactivation measured after 100, 250, and 500 ms depolarization was significantly reduced in Ca_V_2.1-F1800I compared to wildtype controls (Figures 2B–D). For example, in test pulses to +10 mV this resulted in a substantially increased calcium current during the declining phase of the current (Figure 1D). However, analyzing the time constants of inactivation at voltages between 0 mV and +60 mV showed no statistically significant differences (Figure 2E). Furthermore, the analysis of steady-state inactivation demonstrated a reduced availability of Ca_V_2.1-F1800I mutant channels after prolonged depolarizations (Figure 2F). The voltage-dependence of inactivation was shifted by 10 mV to more hyperpolarized potentials, in parallel to the left-shift of the voltage-dependence of activation (Figures 2G,H). Recovery from inactivation after a 5 s depolarization reached about 90% within 45 s, and the time course of recovery was very similar in wildtype and mutant channels (Figures 2I,J). Together, the functional analyses demonstrates that the F1800I substitution in Ca_V_2.1 results in altered channel gating properties with a left-shifted voltage-dependence of activation and inactivation, and somewhat reduced activation and inactivation kinetics (Table 2).

## 4 Discussion

*CACNA1A*-related disorders include a spectrum of distinct clinical phenotypes such as episodic ataxia, familial hemiplegic migraine, and epilepsy, but also overlapping phenotypes with additional symptoms such as developmental delay and cognitive disability. Here, we describe a case of *CACNA1A*-associated disease in a boy with developmental delay and congenital ataxia who developed recurrent status epilepticus and life-threatening postictal apnea after the age of 6 years. A broad spectrum of seizure types has been reported in *CACNA1A*-related epilepsy with status epilepticus often being the initial manifestation. Both gain-of-function and loss-of-function variants have been found in patients with epilepsy (10, 31). However, in patients with status epilepticus mainly gain-of-function variants, located in the transmembrane regions, particularly in segments 4–6, have been reported (8, 9). Accordingly, the novel missense variant p.Phe1800Ile, found in our patient, is located in the transmembrane segment 6 of domain IV (Table 3).

**Table 3:** Rate of recovery from inactivation was measured from a double step protocol using different ∆t (Figure 2I).

	**Ca**_**V**_ **2.1 WT**	**F1800I**	*p*-value
**% of Recovery from inactivation (**∆t / ms**)**			
*n*	7	8	
at 20 ms	8.3 ± 3.7	11.4 ± 2.8	>0.9999
at 35 ms	7.6 ± 3.4	11.5 ± 2.9	0.9996
at 60 ms	8.4 ± 3.1	13 ± 3.1	0.9965
at 100 ms	9.2 ± 3.1	13.3 ± 3.3	0.9992
at 170 ms	10.9 ± 3.2	15.9 ± 3.3	0.9945
at 290 ms	12.8 ± 2.9	20.2 ± 3.9	0.9002
at 490 ms	16.6 ± 3.6	23.9 ± 4.5	0.9783
at 830 ms	21.8 ± 4.1	29.1 ± 5	0.9913
at 1400 ms	26.9 ± 4.8	34.4 ± 6.1	0.9985
at 2400 ms	33.4 ± 5.2	40.9 ± 4.6	0.9982
at 4100 ms	41.8 ± 5.0	47.5 ± 5.6	>0.9999
at 7000 ms	51.4 ± 5.2	54.7 ± 5.5	>0.9999
at 12000 ms	63.8 ± 5.0	64.7 ± 5.3	>0.9999
at 20000 ms	77.1 ± 4.1	76.3 ± 3.9	>0.9999
at 32000 ms	87.1 ± 4.3	86.5 ± 2.8	>0.9999
at 45000 ms	94.1 ± 3.5	95.9 ± 1.5	>0.9999

Mean ± SEM; mutant and WT controls compared by mixed-effects analysis matched with Šídák’s multiple comparisons test, using significance criteria, ∗≜p<0.05,∗∗≜p<0.01,∗∗∗≜p<0.01,∗∗∗∗≜p<0.01.

Functional analysis of the variant in our patient showed that Ca_v_2.1-F1800I channels opened at lower voltages, but also inactivated at lower voltages. Specifically, the left-shifted voltage-dependence of activation and delayed inactivation correspond to a gain of channel function resulting in increased calcium influx during brief depolarizations. Both effects on channel gating of the F1800I mutation in the S6 helix of the fourth repeat are very similar to the electrophysiological effects reported for a deletion variant of the corresponding phenylalanine in the S6 helix of the third repeat of Ca_V_2.1 (ΔF1502) found in patients with congenital ataxia and hemiplegic migraine (32), thus, supporting the causative role of mutations of this highly conserved amino acid in the pore domain of Ca_V_2.1. As Ca_V_2.1 is the major pre-synaptic channel in the central and peripheral nervous system, such altered channel gating would translate in increased neurotransmitter release and synaptic transmission. Whereas Ca_V_2.1 controls neurotransmitter release in both, excitatory and inhibitory synapses, previous studies of gain-of-function Ca_V_2.1 variants revealed enhanced excitatory neurotransmission at glutamatergic pyramidal cell synapses without affecting GABA-ergic neurotransmission at interneuron synapses (33–35). Such a propensity for enhancing excitatory neurotransmission might result in hyperexcitability and thereby explain the seizures in the patients.

The further observed left-shifted voltage-dependence of steady-state inactivation results in a decreased availability of Ca_V_2.1 channels in neurons persistently depolarized to potentials above −30 mV, thus representing a loss-of-function effect of this disease variant. However, under physiological conditions, such persistent depolarizations of neurons are not to be expected and therefore reduced channel availability due to left-shifted voltage-dependence of steady-state inactivation probably is of lesser importance for the pathogenicity of the Ca_V_2.1-F1800I variant. In contrast, the observed gain of channel function could lead to an increased neurotransmitter release probability as well as to synaptic remodeling due to increased calcium influx (36). Although epilepsy has been reported in patients with both gain-of-function variants and loss-of-function *CACNA1A* variants, status epilepticus was more frequently associated with gain-of-function variants (8). Apneic seizures also occurred in a boy with epileptic encephalopathy and the p.Val1808Leu variant, which is located next to the variant found in our patient. However, no functional data are available (37).

Our patient had recurrent status epilepticus occurring during sleep with prolonged central apnea postictally requiring intensive care and implicating a high risk of death without appropriate intervention. In a case review of 130 patients, 6 deaths were reported in children aged 3 months to 5 years with *CACNA1A*-related disease (8). Causes of death were listed as “fatal cerebral edema” and “epileptic encephalopathy” (not referred to as SUDEP) (38, 39). The pathophysiology of SUDEP is assumed to be heterogeneous and not fully understood. Several mechanisms have been discussed, including the spread of cortical depolarization to the brainstem during a seizure and the resulting suppression of cardiorespiratory control (14). SUDEP has a frequency of 1,2/1,000 years of epilepsy patients and higher in patients with generalized tonic–clonic seizures. Several genes, especially channelopathies, have been identified in patients who died of SUDEP including gene variants associated with seizures (e.g., *SCN1A*, *SCN8A*, *SCN2A*) or long QT-Syndrome (*SCN5A*, *KCNH2*, *KCNQ1*) (40–42). Conversely, however, only a few genes have been shown to be associated with an increased SUDEP risk, and it is often unclear whether this is due to a high seizure frequency in these patients or to additional pathophysiological factors caused by the gene variant. Therefore, SUDEP is thought to have a multifactorial origin with a genetic predisposition. In addition to the genes listed above, *CACNA1A* was identified as a potential candidate gene in a cohort of 14 patients who died of SUDEP (43). The *CACNA1A* missense variant p.Ser218Leu is associated with a gain of channel function and in the homozygous *CACNA1A*^218^ mouse, in contrast to the wildtype, seizures led to SUDEP triggered by brainstem spreading depolarization with subsequent apnea and cardiac arrest (44, 45). The shift in the voltage dependence of activation observed here in the Ca_V_2.1-F1800I variant is similar in direction and extent as reported in both neurons of the *CACNA1A^218^* mouse and human recombinant S218L mutant channels (35, 46). Further, Cain et al. (47) demonstrated that the superior colliculi play an important role in the propagation of seizures to the brainstem in *CACNA1A*^218^ mice, leading to fatal seizures. Hyperexcitability of superior colliculus neurons as a result of gain of channel function with lower voltage threshold for calcium influx and prolonged channel opening was speculated by the authors as the underlying mechanism. These data from the *CACNA1A* mouse model closely fit the combination of the gain of channel function observed in our patient, as determined by the functional analysis of our patient’s variant, and the prolonged seizure-related but nonconvulsive apneas leading to multiple life-threatening situations.

In our patient, the combination of levetiracetam plus topiramate plus lamotrigine resulted in the cessation of status epilepticus. Since no selective Ca_v_2.1 channel inhibitors are available, we aimed to modulate channel activity and neuronal calcium homeostasis. To address the gain-of-function in channel activity, we chose the aforementioned unselective calcium channel inhibitors. Different therapeutic approaches in patients with *CACNA1*-related disorder have been discussed, mainly targeting channel activity and its cellular function, such as the use of carbonic anhydrase inhibitors, for example acetazolamide, in both gain- and loss-of-function *CACNA1A* mutations (48–50) as well as non-selective calcium channel blockers or openers to modulate channel activity (51–53). The best benefit of lamotrigine, which acts on the P/Q-type calcium channel, was observed in a patient with epileptic encephalopathy and the *CACNA1A* missense variant p.Ser1373Leu, but functional data on the variant were not reported (54). In the case series of 18 patients, Le Roux et al. (10) found the best efficacy in seizure reduction for topiramate, levetiracetam, lamotrigine, and valproate, which is consistent with the observation in our patient.

In conclusion, patients with *CACNA1A*-related epilepsy are prone to develop status epilepticus. Life-threatening, seizure-related apnea in these patients increases the risk of sudden death in epilepsy and prevention strategies such as pulse oximetry monitoring should be discussed with the families.

## Data Availability

All electrophysiological data presented in the study are included in Tables 2 and 3, further inquiries can be directed to the corresponding author.

## Footnotes

^1^ https://www.ncbi.nlm.nih.gov/snp/

^2^ http://gnomad.broadinstitute.org/
